# *In vivo* genome editing in animals using AAV-CRISPR system: applications to translational research of human disease

**DOI:** 10.12688/f1000research.11243.1

**Published:** 2017-12-20

**Authors:** Cia-Hin Lau, Yousin Suh

**Affiliations:** 1 Department of Mechanical and Biomedical Engineering, City University of Hong Kong, Hong Kong, SAR, China; 2 Department of Genetics, Albert Einstein College of Medicine, Bronx, New York, USA; 3 Department of Ophthalmology and Visual Sciences, Albert Einstein College of Medicine, Bronx, New York, USA; 4 Department of Medicine, Albert Einstein College of Medicine, Bronx, New York, USA; 5 Institute for Aging Research, Albert Einstein College of Medicine, Bronx, New York, USA

**Keywords:** CRISPR/Cas9 complex, genome editing, adeno-associated virus

## Abstract

Adeno-associated virus (AAV) has shown promising therapeutic efficacy with a good safety profile in a wide range of animal models and human clinical trials. With the advent of clustered regulatory interspaced short palindromic repeat (CRISPR)-based genome-editing technologies, AAV provides one of the most suitable viral vectors to package, deliver, and express CRISPR components for targeted gene editing. Recent discoveries of smaller Cas9 orthologues have enabled the packaging of Cas9 nuclease and its chimeric guide RNA into a single AAV delivery vehicle for robust *in vivo* genome editing. Here, we discuss how the combined use of small Cas9 orthologues, tissue-specific minimal promoters, AAV serotypes, and different routes of administration has advanced the development of efficient and precise *in vivo* genome editing and comprehensively review the various AAV-CRISPR systems that have been effectively used in animals. We then discuss the clinical implications and potential strategies to overcome off-target effects, immunogenicity, and toxicity associated with CRISPR components and AAV delivery vehicles. Finally, we discuss ongoing non-viral-based *ex vivo* gene therapy clinical trials to underscore the current challenges and future prospects of CRISPR/Cas9 delivery for human therapeutics.

## Introduction

CRISPR (clustered regulatory interspaced short palindromic repeat)/Cas9-based RNA-guided DNA endonuclease is transforming biomedical science research and has quickly become the preferred genome-editing platform for interrogating endogenous gene function *in vivo* ^1, 2^. The CRISPR-based genome-editing tool has revolutionized the gene-editing technique because of its simplicity in target design, affordability, high efficiency, versatility, and multiplexing capability ^3^. The commonly used CRISPR system can be implemented in mammalian cells by co-expressing Cas9 nuclease along with chimeric guide RNA (gRNA), which is derived from a synthetic fusion of the CRISPR RNA array (crRNA) and trans-activating crRNA (tracrRNA) ^3^. The target site sequence of gRNA needs to be immediately followed by an optimal protospacer adjacent motif (PAM) sequence according to the species-derived Cas9 nuclease used at the 3′ end ^4, 5^. The background of and the recent developments in the CRISPR-based targeted genome-editing toolboxes have been extensively reviewed recently ^1, 2, 6^. Various applications of CRISPR technologies for genome engineering and medical research have also been reviewed recently ^2, 6, 7^.

The CRISPR/Cas9 complex can be introduced into the cell in the forms of DNA, messenger RNA (mRNA), or protein ^8^. The DNA encoding Cas9 and gRNA can be delivered into the cell using the plasmid and viral expression vectors ^8^. Through microinjection, liposome-mediated transfection, electroporation, or nucleofection ^9^, several recent studies have shown that the delivery format of active Cas9 protein/gRNA ribonucleoprotein (RNP) complex has lower off-target effects due to rapid clearance of RNPs from the cell ^8– 10^ and rapid gene editing as they cleave chromosomal DNA almost immediately after delivery ^9, 10^ as compared with plasmid DNA transfection. However, the delivery formats of mRNA and protein pose certain technical challenges *in vivo*. For example, many of the genetic brain disorders affect very large brain areas and more than a single structure in the brain ^11^. Therefore, global gene delivery to the central nervous system is a key to achieve effective therapies for neurological disorders. Although a recent study has successfully demonstrated genome editing in the mouse brain by local delivery of RNPs ^12^, it is technically challenging to deliver the RNPs globally to the central nervous system. Thus, the viral-based *in vivo* genome editing remains a popular choice to achieve stable or elevated expression of Cas9 and single-guide RNA (sgRNA) required for establishing animal disease models and therapeutic gene editing in animals. Indeed, it has been demonstrated that systemic delivery of adeno-associated virus (AAV) vectors enabled uniform and broad vector distribution, and subsequently led to extensive and widespread transgene expression in the adult mouse central nervous system ^13– 15^.

Animal models are preferred over cell models, as they help understand disease mechanisms at the physiological and systemic levels. Somatic mutagenesis via *in vivo* genome editing provides an ideal platform to accelerate the generation of transgenic animals for rapid exploration of human diseases and correcting genetic defects in gene therapy. *In vivo* genome editing also avoids laborious germline targeting and high costs of maintaining transgenic neonates through adulthood in animals with a long life span. Obviously, somatic mutagenesis in adult animals has proven technically more challenging than gene editing of one-cell-stage embryos or zygotes or pluripotent stem cells. The major hurdle is to efficiently deliver the CRISPR components *in vivo*. To date, various viral, non-viral (for example, lipid nanoparticles), and physical (for example, hydrodynamic injection) based delivery approaches of the CRISPR/Cas9 complex have been adopted for *in vivo* genome editing. The pros and cons of these delivery methods have been extensively reviewed recently ^16– 18^.

Given the great potential of viral vectors in gene and cell therapy, five major classes of viral vectors—retroviruses ^19^, lentiviruses ^20, 21^, adenoviruses ^22, 23^, AAVs ^24, 25^, and baculoviruses ^26, 27^—have been employed to deliver CRISPR components into mammalian cells for targeted genome editing. The advantages and disadvantages of using these viral vectors for *in vivo* delivery of the CRISPR transgenes have been extensively reviewed ^24, 28– 30^. In Table 1, we listed general characteristics and applications of various viral delivery vectors. Among these, the AAV vector is the overall focus of this review. The AAV system provides major advantages for research and therapeutics, including a very mild immune response and toxicity elicited by AAV in animal models. Moreover, AAVs remain primarily episomal upon transduction, avoiding random integration of the viral genetic materials into the host genome that can disrupt neighboring gene function and cause insertional mutagenesis ^31^. Indeed, there has been no reported case of disease caused by AAV in humans ^31^. Additionally, AAV can exist long-term as concatemers in non-dividing cells for stable transgene expressions ^31^. Given a good safety profile of AAV and therapeutic efficacy in a wide range of animal models and human clinical trials (ClinicalTrials.gov), AAV is thought to be one of the most suitable viral vectors for gene therapeutic applications and gene transfer *in vivo*. Furthermore, there is a wide range of AAV serotypes that can be selected to infect specific tissues *in vivo*. For these reasons, here, we provide an overview of the state of the art of various AAV-CRISPR systems as well as their principal vector designs for *in vivo* genome editing in animals. Senís and colleagues were amongst the first to exploit and demonstrate the use of the AAV vectors to package, deliver, and express CRISPR components for targeted gene editing in hard-to-transfect cells and the liver of adult mice ^32^. Subsequently, an increasing number of the studies used AAV vectors to deliver the CRISPR components into animals for *in vivo* genome editing. Of note, for certain applications such as packaging of a very large transgene ^29, 33^, genome-wide screening ^20, 34, 35^, and production of vaccines and recombinant proteins ^36, 37^, the other viral vectors may be a better option. However, in-depth discussions of the other viral delivery vehicles for the CRISPR components are beyond the scope of this review.

**Table 1.** Viral delivery vectors for the CRISPR/Cas9 complex.

Characteristics for a typical vector	Retrovirus ^19, 38, 39^	Lentivirus ^20, 21, 38, 40, 41^	Adenovirus ^22, 42– 48^	Adeno-associated virus ^30, 32, 49– 52^	Baculovirus ^27, 29, 53^
Common viral type	γ-retroviruses	HIV-1	Ad5	AAV2	AcMNPV
Viral envelope	Yes	Yes	No	No	Yes
Nucleocapsid shape	Icosahedral	Icosahedral	Icosahedral	Icosahedral	Rod
Viral size	80–130 nm	80–130 nm	70–105 nm	18–26 nm	250–300 nm 30–60 nm
Viral genome structure	Linear ssRNA	Linear ssRNA	Linear dsDNA	Linear ssDNA	Circular dsDNA
Viral genome size	8.3 kb	9.7 kb	36 kb	4.7 kb	80–180 kb
Packaging capacity	<8.0 kb	<8.0 kb	<30 kb	<4.5 kb	>38 kb
Transgene is flanked by	LTRs	LTRs	ITRs	ITRs	Tn7s
Viral generation approach	Triple-plasmid transfection	Triple-plasmid transfection	Homologous recombination	Triple-plasmid transfection	Site-specific transposition
Competent cell used	Stbl3	Stbl3	AdEasier-1	Stbl3	DH10Bac
Host cells used	HEK293T	HEK293T	HEK293T or HER911	HEK293T	Sf9 or Sf21
Cells infected	Dividing	Dividing or non-dividing	Dividing or non-dividing	Dividing or non-dividing	Dividing or non-dividing
Transduction efficiency	Moderate	High	Very high	High	High
Transgene expression	Stable	Stable	Transient	Transient	Transient
Immune response	Moderate	Low	High	Very low	Very low
Toxicity	High	Moderate	High	Low	Low
Random genome integration	Yes	Yes	No	Generally no (recombinant AAV has a low frequency of host genome integration events)	No
Biosafety levels	BSL-2	BSL-2	BSL-2	BSL-1	BSL-1
Common applications	Generating stable cell and gene transfer, cancer and stem cell research	Transduce difficult-to- transfect cell, genome- wide screens	Vaccine production, cancer immune therapy	Gene delivery *in vivo*, optogenetics	Recombinant proteins and vaccine production
Clinical trials	Very popular	Very popular	Popular	Increasing popularity	Growing interest
First *ex vivo* gene transfer clinical trial started (disease, ClinicalTrials.gov ID)	1990 (severe combined immunodeficiency, NCT00001255)	2007 (lymphoma, NCT00569985)	2000 (hepatocellular carcinoma, NCT00300521)	None	None
First *in vivo* gene transfer clinical trial started (disease, ClinicalTrials.gov ID)	None	2014 (sickle cell anemia, NCT02186418)	1993 (cystic fibrosis, NCT00004779)	1999 (cystic fibrosis, NCT00004533)	None

AcMNPV, autographa californica multicapsid nucleopolyhedrovirus; HIV, human immunodeficiency virus; ITR, inverted terminal repeat; LTR, long terminal repeat; Tn7, transposon 7.

Here, we aim to review various AAV-CRISPR systems recently demonstrated in mice and to discuss how the combined use of tissue-specific minimal promoters, AAV serotypes, different routes of administration, and small Cas9 orthologues has enabled investigators to achieve maximal efficiency and specificity for *in vivo* genome editing. In addition, we discuss the clinical implications and potential strategies to overcome off-target effects, immunogenicity, and toxicity associated with CRISPR components and AAV delivery vehicles. Finally, we discuss the promises and hurdles associated with ongoing *ex vivo* gene therapy clinical trials.

## AAV-CRISPR-based *in vivo* genome editing in mice

At least 33 published studies successfully used CRISPR/Cas9 alongside AAV vectors for *in vivo* genome editing in mice ( Table 2). The AAV-CRISPR system is particularly useful for editing disease-associated genes in the brain or central nervous system of mice because of the inability of most cationic nanocarriers to cross the blood-brain barrier, high transduction efficiency of AAV vectors in the brain, and non-dividing properties of neurons for long-term therapeutic effects. The AAV-CRISPR system has also been successfully used to restore the gene function in muscle-associated diseases such as Duchenne muscular dystrophy ^54– 57^ and diseases associated with the eye ^5, 58– 60^, liver ^4, 49, 61^, heart ^62– 64^, and lung ^65^. This AAV-CRISPR-mediated gene editing in mice provided proof-of-principle studies for human disease modeling, gene therapy, or gene functional characterizations. We discuss recent developments and advancements in four critical aspects of the AAV-CRISPR system that have enabled efficient and precise *in vivo* genome editing in mice.

**Table 2.** CRISPR/Cas9-based *in vivo* genome editing in mice with tissue-specific promoters and AAV variants.

Target tissue in mouse (disease or phenotype)	Promoter – regulates CRISPR or reporter	AAV serotype	Route of administration (injection site)	Application (gene)	Phenotypic impact or therapeutic outcome	Remark	Reference
Brain (study gene function)	pMecp2 promoter - SpCas9 hSyn1 promoter - GFP, KASH U6 promoter - gRNA	AAV1	Stereotactic injection (dentate gyrus)	Gene knockout ( *Mecp2*, *Dnmt1*, *Dnmt3a*, and *Dnmt3b*)	Impaired contextual memory and memory formation	Can edit multiple genes simultaneously	25
Brain (study brain circuit)	hSyn1 promoter - SaCas9, Cre recombinase, GCaMP6f calcium sensor CAG promoter - tdTomato U6 promoter - Grna	AAV2-retro	Stereotactic injection (pontine nucleus, dorsal striatum)	Gene knockout ( *tdTomato*)	Enabled efficient mapping, monitoring, and manipulation of projection neurons	Modified AAV2 capsid with a 7-mer peptide	68
Brain (Huntington disease)	CMV promoter - SpCas9, eGFP U6 promoter - gRNA H1 promoter - gRNA	AAV1	Stereotactic injection (striata)	SNP- dependent editing ( *Htt*)	Reduced expression of mutant *Htt* allele in mouse brain	Use transgenic Huntington disease model	76
Brain (schizophrenia)	EF1a promoter - tdTomato CBh promoter - ScGFP hSyn1 promoter - ScGFP GFAP promoter - ScGFP U6 promoter - gRNA	AAV2g9	Stereotaxic injections (intracerebroventricular, cerebrospinal fluid)	Gene deletion ( *pre-miR137*)	Displayed preferential, robust, and widespread neuronal transduction within the brain	Use Cas9 mice and an AAV chimeric derived from AAV2 and AAV9	80
Brain (inducible genome editing)	Dox inducible Tight promoter - SpCas9 TRE3G promoter - SpCas9 pMecp2 - SpCas9 CMV promoter - TetR, GFP, KASH H1/TO promoter - gRNA U6/TO promoter - gRNA	AAV-DJ AAV-DJ/8	Stereotaxic injection (basal and lateral amygdala)	Gene induction ( *Tet2*)	Edited the genomes of neurons *in vivo* within the mouse brain in a Dox- dependent manner	Doxycycline- dependent gRNA expression	81
Brain (study gene function)	CBh promoter - Cre recombinase hSyn1 promoter - GFP, KASH U6 promoter - gRNA	AAV1	Stereotactic injection (prefrontal cortex)	Gene knock-in ( *NeuN*)	NeuN protein depletion only in the injected region	Use Cre-dependent SpCas9 *Rosa26* knock-in mice	65
Brain (glioblastoma)	U6 promoter - gRNA GFAP promoter - Cre recombinase	AAV9	Stereotactic injection	Gene mutation ( *Trp53*, *Nf1*, or *Rb1*)	Induced tumor formation that recapitulates human glioblastoma	Use LSL-Cas9 mice	79
Brain (precise genome editing)	EFS promoter - SpCas9 U6 promoter - gRNA	AAV1	Intraventricular and stereotactic injections	Gene knock-in ( *Camk2a*, *Erk2*, *Actb*)	vSLENDR enabled efficient homology-directed repair in post-mitotic neurons in developing, adult, aged, and pathological brains	Use wild-type and Cas9 mice	82
Brain (study gene function)	U6 promoter - gRNA CBh promoter - Cre recombinase	AAV	Stereotactic injection (pyramidal neurons and microglia in hippocampus)	Gene disruption ( *Cnr2*)	Decreased contextual fear memory, enhanced spatial working memory	Use Camk2a-Cas9, Gad2-Cas9, and Cx3cr1-Cas9 mice	83
Central nervous system (amyotrophic lateral sclerosis)	CMV promoter - SpCas9 U6 promoter - gRNA	scAAV9	Intrathecal injection	Gene knockdown ( *Igf1*)	Decreased D-amino acid oxidase and increased D-serine, and caspase9 activation	Use hSOD1G93A ALS mouse model	84
Muscle (Duchenne muscular dystrophy)	CMV promoter - SaCas9 EFS promoter - SaCas9 CAGGS promoter - tdTomato U6 promoter - gRNA	AAV9	Intramuscular (tibialis anterior) and intraperitoneal (intraperitoneal space) injections	Exon deletion ( *Dmd*)	Partially recovered muscle functional deficiencies	Use mdx mouse model of DMD	54
Muscle (Duchenne muscular dystrophy)	CMV promoter - SaCas9 U6 promoter - gRNA	AAV8	Intramuscular (tibialis anterior), intraperitoneal (intraperitoneal space), and intravenous (tail vein) injections	Exon deletion ( *Dmd*)	Improved muscle function	Use mdx mouse model of DMD	55
Muscle (Duchenne muscular dystrophy)	CMV promoter - SpCas9 U6 promoter - gRNA	AAV9	Intraperitoneal, intramuscular, and retro-orbital (venous sinus) injections	Exon deletion ( *Dmd*)	Enhanced skeletal muscle function	Use mdx mouse model of DMD	56
Muscle (Duchenne muscular dystrophy)	CK8 promoter - SpCas9, SaCas9 CMV promoter - mCherry U6 promoter - gRNA	AAV6	Intramuscular (tibialis anterior) and systemic (retro-orbital) injection	Exon deletion and gene knock-in ( *Dmd*)	Improved muscle function	Use mdx4cv mouse model of DMD	57
Muscle (congenital muscular dystrophy type 1A)	CMV promoter - SaCas9 U6 promoter - gRNA	AAV9	Intraperitoneal injection	Gene correction ( *Lama2*)	Improved muscle histopathology and function	Use dy2J/dy2J mice	69
Retina (age-related macular degeneration)	EFS promoter - CjCas9 Spc512 promoter - CjCas9 U6 promoter - gRNA	AAV9	Intramuscular (tibialis anterior) and intravitreal injection	Gene knockout ( *Rosa26*, *Vegfa*, and *Hif1a*)	Reduced the size of laser-induced choroidal neovascularization	Smallest Cas9 orthologue (smaller than SaCas9)	5
Retina (Leber Congenital Amaurosis 10)	CMV promoter - SpCas9, SaCas9 hSyn1 promoter - eGFP GFAP promoter - eGFP U6 promoter - gRNA	AAV5	Subretinal injection	Gene deletion ( *Cep290*)	Effectively removed intronic mutation in *Cep290* with minimized immune response	Use self-limiting CRISPR/Cas9 system	58
Retina (retinal gene editing)	pMecp2 promoter - SpCas9 hSyn1 promoter - mCherry, KASH U6 promoter - gRNA	AAV2	Intravitreal (intraocular) injection	Gene knockout ( *YFP*)	Effective gene knockout without affecting retinal function	Use Thy1-YFP transgenic mice	59
Retina (retinal degeneration)	CMV promoter - SpCas9, tdTomato U6 promoter - gRNA	AAV8	Subretinal injection	Gene knockout ( *Nrl*)	Prevented retinal degeneration, improved rod survival, and preserved cone function	Use mouse models of retinal degeneration	60
Retina (angiogenesis)	pICAM2 - SpCas9 U6 promoter - gRNA	AAV1	Intravitreal injection	Gene silencing ( *VEGFR2*)	Abrogated angiogenesis	Use mouse models of oxygen-induced retinopathy and laser-induced choroid neovascularization	85
Liver (ornithine transcarbamylase)	TBG promoter - SaCas9 U6 promoter - gRNA	AAV8	Intravenous injection (temporal vein)	Gene knock-in ( *Otc*)	Increased survival in mice challenged with a high-protein diet	Use adult OTC- deficient mice	49
Liver (hereditary tyrosinemia)	EF1a promoter - GFP U6 promoter - gRNA	AAV8	Systemic (tail vein) injection	Gene knock-in ( *Fah*)	Rescued disease symptoms such as weight loss and liver damage	Use a mouse model of human hereditary tyrosinemia	61
Liver (total cholesterol)	TBG promoter - SaCas9 U6 promoter - gRNA	AAV8	Systemic (tail vein) injection	Gene knockout ( *Pcsk9*)	Reduced serum Pcsk9 and total cholesterol levels	Smaller Cas9 orthologue than SpCas9	4
Liver (LDL cholesterol)	CB promoter - EmGFP U6 promoter - gRNA	AAV8	Intraperitoneal injection	Gene knockout ( *Ldlr*, *Apob*)	Resulted in severe hypercholesterolemia and atherosclerosis	Use Cas9 targeted transgenic mice	77
Liver (hemophilia B)	HCRhAATp promoter - SaCas9 U6 promoter - gRNA	AAV8	Systemic (tail vein) injection	Gene knock-in ( *F9*)	Restored hemostasis	Use *F9*-mutated mice	71
Heart (PRKAG2 cardiac syndrome)	CMV promoter - SpCas9 U6 promoter - gRNA	AAV9	Systemic injection	Gene knockout ( *Prkag2*)	Restored the morphology and function of the heart after disrupting the mutant *Prkag2* allele	Use H530R *Prkag2* transgenic knock-in mice	62
Heart (cardiac disease modeling)	Myh6 promoter - SpCas9, GFP, TdTomato CMV promoter - ZsGreen U6 promoter - gRNA	AAV9	Intraperitoneal injection	Gene deletion ( *Myh6*)	Displayed severe cardiomyopathy and loss of cardiac function	Use postnatal cardiac-Cas9 transgenic mice	63
Heart (cardiac myocyte maturation)	cTNT promoter - Cre recombinase U6 promoter - gRNA	AAV9	Subcutaneous injection	Gene knockout ( *Jph2*, *Ryr2*)	Disrupted T-tubule structure and maturation	Use RosaCas9GFP/ Cas9GFP neonatal mice	78
Heart (dystrophic cardiomyopathy)	CK7-miniCMV promoter - SaCas9 U6 promoter - gRNA	AAV rh74	Systemic (retro-orbital, intraperitoneal) injection	Gene excision ( *Dmd*)	Restored dystrophin expression and cardiac function	Use mdx/Utr ^+^/ ^−^ dystrophic mice	70
Heart (cardiac gene function)	CB promoter - SpCas9 U6 promoter - gRNA	AAV9	Intracardiac	Gene disruption ( *Myh6*, *Sav1*, and *Tbx20*)	Resulted in mosaic pattern of gene disruption	Use Myh6-Cre transgenic mice	64
Lung (lung adenocarcinoma)	EFS promoter - Renilla luciferase, Cre recombinase U6 promoter - gRNA	AAV9	Intranasal (nostril) and intratracheal (trachea) injections	Gene knock-in ( *p53*, *Lkb1*, and *Kras*)	Led to macroscopic tumors of lung adenocarcinoma pathology	Use Cre-dependent SpCas9 *Rosa26* knock-in mice	65
Liver, heart, muscle (host immune response)	SMVP promoter - SpCas9, SpCas9-VPR CASI promoter - SpCas9 CAG promoter - tdTomato U6 promoter - gRNA	AAV9	Intramuscular and intraperitoneal injections	Gene activation ( *Mstn*, *Fst*, *Pd-l1*, and *Cd47*)	Modest activation of *Pd-l1* and *Cd47*	Use AAV-split-Cas9 system	50
Circulating lymphocytes, spleen, liver, heart, lung, and kidney (eradication of HIV-1 DNA)	CMV promoter - SaCas9 U6 promoter - gRNA	AAV9	Systemic (tail vein) injection and retro- orbital inoculation	Gene knockout (HIV-1 DNA)	Eradication of HIV-1 DNA	Use transgenic mice and rats encompassing the HIV-1 genome	86
Spleen, lungs, heart, colon, and brain (HIV-1 proviral DNA excision)	CMV promoter - SaCas9 U6 promoter - gRNA	AAV-DJ/8	Systemic (tail vein) injection	Gene deletion (HIV-1 DNA)	Induced efficient excision of HIV-1 proviral DNA	Use HIV-1 Tg26 transgenic mice and humanized BLT mice with chronic HIV-1 infection	72

AAV, adeno-associated virus; ALS, amyotrophic lateral sclerosis; *Apob*, apolipoprotein B; BLT, bone marrow/liver/thymus; CAG, a hybrid of CMV early enhancer and chicken beta-actin promoter; CAGGS, a hybrid of promoter composed of the CMV immediate-early enhancer, CBA promoter, and CBA intron 1/exon 1; *Camk2a*, calcium/calmodulin-dependent protein kinase II alpha; CASI, a hybrid of CMV enhancer and chicken β-actin promoter followed by a splice donor and splice acceptor; CB, chicken beta actin promoter; CBh, a hybrid form of the CBA promoter; *Cd47*, integrin-associated signal transducer; *Cep290*, centrosomal protein 290; CjCas9, *Campylobacter jejuni* Cas9; CK7, CK8, striated muscle-restricted promoter; CMV, cytomegalovirus; *Cnr2*, cannabinoid receptor 2; CRISPR, clustered regularly interspaced short palindromic repeat; cTNT, cardiac troponin T promoter; *Cx3cr1*, chemokine (C-X3-C motif) receptor 1; *Dmd*, Duchenne muscular dystrophy; *Dnmt*, DNA methyltransferase; EF1a, elongation factor-1 alpha; EFS, EF1a short; eGFP, enhanced green fluorescent protein; *Erk2*, mitogen-activated protein kinase 1; *F9*, coagulation factor IX; *Fah*, fumarylacetoacetate hydrolase; *Fst*, follistatin; *Gad2*, glutamic acid decarboxylase 2; GCaMP6f, green fluorescent calcium indicator; GFAP, glial fibrillary acidic protein; H1, human polymerase III RNA promoter; HCRhAATp, an enhancer element of the hepatic control region of the Apo E/C1 gene and the human anti-trypsin promoter; *Hif1a*, hypoxia-inducible factor 1 alpha; HIV, human immunodeficiency virus; hSyn1, human synapsin I; *Htt*, huntingtin; *Igf1*, insulin-like growth factor 1; *Jph2*, junctophilin-2; KASH, nuclear transmembrane domain; *Kras*, Kirsten rat sarcoma viral oncogene homolog; *Lama2*, laminin alpha 2; LDL, low-density lipoprotein; *Ldlr*, low-density lipoprotein receptor; *Lkb1*, serine/threonine kinase 11; mdx, dystrophin-deficient; *Mecp2*, methyl CpG binding protein 2; *Mstn*, myostatin; *Myh6*, myosin heavy polypeptide 6; *NeuN*, neuronal nuclear antigen; *Nf1*, neurofibromin 1; *Nrl*, neural retina-specific leucine zipper protein; *Otc*, ornithine transcarbamylase; *Pcsk9*, proprotein convertase subtilisin/kexin type 9; *Pd-l1*, programmed death-ligand 1; pICAM2, intercellular adhesion molecule 2 promoter; *Prkag2*, protein kinase AMP-activated non-catalytic subunit gamma 2; *p53*, transformation related protein 53; *Rb1*, RB transcriptional corepressor 1; *Roa26*, a safe harbor locus in mouse; *Ryr2*, ryanodine receptor 2; SaCas9, *Staphylococcus aureus* Cas9; *Sav1*, salvador family WW domain containing 1; SMVP, a hybrid of SV40 enhancer–CMV–promoter–chimeric intron; SNP, single-nucleotide polymorphism; SpCas9, *Streptococcus pyogenes* Cas9; Spc512, muscle-specific promoter; TBG, thyroxine binding globulin; *Tbx20*, T-box 20; *Tet2*, tet methylcytosine dioxygenase 2; *Thy1*, thymus cell antigen 1 theta; TRE3G, Tet-On 3G inducible promoter; TO, tetracycline operator; U6, human U6 small nuclear promoter; *Vegfa*, vascular endothelial growth factor A; *VEGFR2*, kinase insert domain protein receptor; VPR, a fusion of VP64–p65–Rta; vSLENDR, virus-mediated single-cell labeling of endogenous proteins via HDR; YFP, yellow fluorescent protein.

### Small Cas9 orthologues

**Figure 1.**
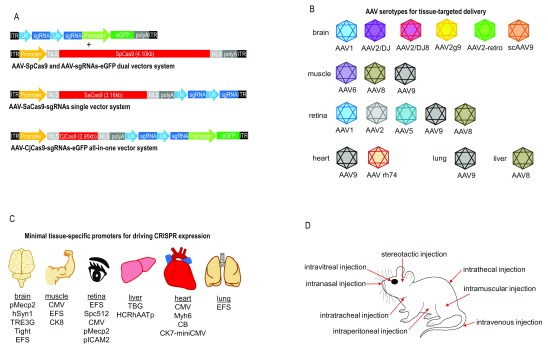
CRISPR/Cas9-based *in vivo* genome editing by using small Cas9 orthologues, different routes of AAV administration, tissue-specific minimal promoters, and AAV serotypes. ( **A**) AAV-CRISPR vectors. *Campylobacter jejuni*-derived Cas9 (CjCas9) is the smallest Cas9 orthologue (984 amino acids, 2.95 kb). *Staphylococcus aureus*-derived Cas9 (SaCas9) (1,053 amino acids, 3.16 kb) is smaller than the most widely used Cas9 derived from *Streptococcus pyogenes* (SpCas9) (1,368 amino acids, 4.10 kb). Owing to the large size of SpCas9, the SpCas9 gene and two gRNAs are packaged into two separate AAV vectors. The SpCas9-based dual-vector system enables one vector to express SpCas9 and another to express multiple gRNAs and a fluorescent reporter gene. Two gRNAs can be packaged with the SaCas9 into a single AAV vector if minimal promoter and polyadenylation signal are used. In the all-in-one vector system, CjCas9 enables packaging with two gRNAs and a fluorescent reporter gene, enhanced green fluorescent protein ( *eGFP*), into a single AAV vector. ( **B**) AAV serotypes for tissue-targeted delivery. ( **C**) Tissue-specific minimal promoters for driving CRISPR expression. ( **D**) Different routes of AAV administration into various tissues of a mouse. AAV, adeno-associated virus; CRISPR, clustered regulatory interspaced short palindromic repeat; gRNA, guide RNA.

Even though the AAV vector is an extremely attractive delivery vehicle for CRISPR, its relatively small viral genome-packaging capacity has limited its use for delivering large transgenes. Using dual-vector AAV system or smaller Cas9 orthologues along with truncated regulatory elements (promoter and polyadenylation signal) has greatly circumvented the transgene packaging issue ( Figure 1A). Owing to a small AAV viral genome-packaging capacity (~4.8 kb), it has been technically challenging to co-package *Streptococcus pyogenes*-derived Cas9 (SpCas9) (4.1 kb) and multiple sgRNAs into all-in-one AAV vectors for multiplex genome editing. Recently, it has been demonstrated that when very small promoters were used (for example, miCMV promoter and H1 promoter to drive the expressions of SpCas9 and its gRNA, respectively), it was possible to package both SpCas9 and its gRNA into a single vector ^32^. However, the number of promoters that can be selected for tissue-specific transduction is limited. To circumvent this issue, the previously reported dual-vector system was adopted to express SpCas9 nuclease from one vector and to express its gRNAs together with a fluorescent reporter gene from another vector ^25^. Recently, St1Cas9 (~3.3 kb) from *Streptococcus thermophilus* ^66^ and a rationally designed truncated form of SpCas9 ^67^ were developed to fit SpCas9 and its gRNA into a single AAV construct. Unfortunately, St1Cas9 requires a more complex PAM sequence that limits the number of targetable loci ^66^, and truncated SpCas9 has a much lower efficiency than its wild-type counterpart ^67^.

To overcome these drawbacks, other recently discovered small Cas9 orthologues, including *Staphylococcus aureus*-derived Cas9 (SaCas9, 3.16 kb) ^4^ and *Campylobacter jejuni*-derived Cas9 (CjCas9, 2.95 kb) ^5^, have been used to package the Cas9 and its gRNA into a single AAV delivery vehicle for *in vivo* genome editing. To date, at least 11 independent *in vivo* studies have used the AAV-SaCas9 system to edit disease-associated genes in a variety of tissues, including brain ^68^, muscle ^54, 55, 57, 69^, retina ^58^, heart ^70^, and liver ^4, 49, 71^. More recently, the quadruplex gRNAs/SaCas9 vector consisting of SaCas9 and multiplex sgRNAs was successfully delivered using AAV-DJ/8 for *in vivo* excision of HIV-1 proviral DNA in various solid tissues/organs via a single intravenous injection in humanized bone marrow/liver/thymus (BLT) mice with chronic HIV-1 infection ^72^. In the all-in-one vector system, CjCas9 can be packaged with multiple gRNAs and even a fluorescent reporter gene into a single AAV vector ^5^. Even though CjCas9 represents the smallest Cas9 orthologue characterized to date, only one *in vivo* study has demonstrated the use of AAV vectors to deliver CjCas9 into the mouse’s retina for the treatment of age-related macular degeneration because of the recent development of CjCas9 ^5^. Excitingly, AAV-CjCas9 offers the possibility of editing multiplex genes and tracking the expression of fluorescent reporter genes at high efficiency by simply introducing a single type of AAV vector into the mouse body. The CRISPR-mediated genome-editing specificities can be further improved by adopting truncated gRNAs ^73^ and small chemical molecules to regulate Cas9 activity ^74^ or to enhance double-stranded break-induced homologous recombination efficiency ^75^ without sacrificing on-target genome-editing efficiencies.

### Natural and engineered AAV serotypes

Multiple naturally occurring AAV serotypes have been harnessed to deliver the CRISPR/Cas9 complex into different tissues and organs in mice ( Figure 1B). AAV1 is particularly efficient to drive CRISPR transgene expression in the brain ^25, 65, 76^, whereas AAV8 appears well suited for the transduction of liver ^4, 49, 61, 71, 77^ and muscle ^55^ in mice. AAV9 has the general ability to transduce all major tissues, including muscle ^54, 56, 69^, retina ^5^, heart ^62– 64, 78^, and lung ^65^, in mice. Recently, the AAV9-CRISPR system has been successfully used to identify functional tumor suppressors in glioblastoma by performing high-throughput mutagenesis in the brain of conditional-Cas9 mice to recapitulate human glioblastoma ^79^. AAV2 ^59^ and AAV5 ^58^ have also been used to transduce retina, whereas AAV6 ^57^ enables CRISPR-mediated gene editing in muscle tissue.

In order to alter tropism, reduce blockage by neutralizing antibodies, or enhance transduction efficiency, AAV capsids have been chemically or genetically modified to produce hybrid capsids by combining the properties of multiple serotypes, capsid shuffling, directed evolution, rational mutagenesis, or carrying peptide insertions that introduce the novel receptor-binding activity. AAV variants with engineered capsids (AAV2-retro, AAV2g9, AAV-DJ, and AAV-DJ/8) have been used to package CRISPR for transduction in the mouse brain. An *in vivo*-directed evolution-engineered AAV variant, rAAV2-retro, permits robust retrograde access to projection neurons in functionally connected and highly distributed networks with a high efficiency for neural circuit dissection and *in vivo* genome editing ^68^. An AAV chimera derived from AAV2 and AAV9, AAV2g9, enables preferential, robust, and widespread neuronal transduction within the mouse brain for CRISPR/Cas9-mediated gene deletion for the treatment of neurological disorders ^80^. To provide substantially higher infectivity rates across a broad range of tissues than any known native serotypes, the AAV-DJ was engineered by DNA family shuffling to create a hybrid capsid from eight different native serotypes ^87^. AAV-DJ/8 was created by introducing two point mutations in the heparin-binding domain of AAV-DJ to increase uptake in brain tissue *in vivo*, leading to a similar ability to infect heart and brain tissues with AAV8 and AAV9 ^87, 88^. In fact, AAV-DJ/8 has been successfully used alongside the CRISPR/Cas9 system with doxycycline-dependent gRNA expression for inducible genome editing of neurons *in vivo* within the mouse brain ^81^. AAV-DJ/8 has also been successfully used to package and deliver the CRISPR/SaCas9 vectors for *in vivo* excision of HIV-1 proviral DNA in animal models ^72^.

Recently, the capsids of AAV9, which is the most efficient native serotype characterized to date for *in vivo* transduction of the brain, have been further engineered for better performance in transductions and tissue targeting specificity. These include AAV-PHP.B (a chimeric AAV9 variant generated by inserting a 7-mer sequence, TLAVPFK, on AAV9 capsid) ^13^, AAV-AS (a chimeric AAV9 variant generated by inserting a poly-alanine peptide on AAV9 capsid) ^14^, and AAV9/3 (double tyrosine-mutant form of AAV9) ^89^, all of which were shown to be more efficient in transductions of the adult mouse central nervous system after intravenous injection compared with the wild-type AAV9. Furthermore, some of these engineered AAV capsids could de-target the native AAV tropism without affecting vector production, capsid assembly, infectivity, and gene transfer. Therefore, these novel engineered vectors offer promising options to package the CRISPR/Cas9 complex to efficiently edit disease-associated genes in the mouse brain or any other tissues. Additionally, self-complementary AAV9 (scAAV9) can be used to significantly decrease the time required for the onset of CRISPR expression in the central nervous system of mice ^84^. Second-strand synthesis is a rate-limiting step for efficient transduction by recombinant AAV vectors ^31^. The use of double-stranded self-complementary AAV (scAAV) has improved the transduction efficiencies by bypassing the second-strand synthesis step, but cloning capacity was further reduced to accommodate the complementary strand ^90^.

### Tissue-specific minimal promoters

A tissue-specific promoter is important to ensure the expression of the CRISPR transgene in a tissue- or organ-specific manner. Various truncated or minimal tissue-specific promoters have been used to drive the expression of CRISPR in specific tissues upon injection of the AAV vectors into the mouse body ( Figure 1C). For example, the 235 base pair (bp) mouse pMecp2 (methyl CpG binding protein 2) and 485 bp human hSyn1 (human synapsin I) promoters have been used to efficiently and specifically drive the expression of CRISPR and other transgenes in the mouse brain ^25^ and retina ^59^. A striated muscle-restricted promoter, CK8, enabled specific expression of SpCas9 and SaCas9 for gene editing in the muscle of the mdx mouse model of Duchenne muscular dystrophy upon intramuscular (tibialis anterior) or systemic (retro-orbital) injection of AAV6 vectors ^57^. The liver-specific TBG (thyroxine binding globulin) ^4, 49^ and cardiac-specific Myh6 (myosin heavy polypeptide 6) ^63^ promoters have been successfully used to drive the expression of CRISPR for *in vivo* genome editing in mouse liver and heart, respectively. More general promoters such as CMV (cytomegalovirus) ^54, 58, 60, 62^ and EFS (elongation factor-1 alpha short) ^5, 65^ can be used to express the CRISPR in multiple tissues, including muscle, retina, heart, and lung, for simultaneous gene editing of various tissues in mice. Compared with the CMV promoter, the elongation factor-1 alpha (EF1a) promoter tends to give more consistent and prolonged expression regardless of cell type or physiology ^91, 92^. EF1a is more stable in long-term culture because it maintains high transgene expression and stability and the copy number of episomal vectors ^91^. Thus, despite a stronger gene expression driven by the CMV promoter in many mammalian cell types, EF1a is preferred for efficient transgene expression in mouse embryonic stem cell lines ^93^ and for engineering stable tumor cell lines ^92^. The library of minimal tissue-specific promoters alongside AAV vectors used for controlling the expression of CRISPR or other transgenes *in vivo* is expanding with the development of more tightly regulated synthetic hybrid promoters, such as widely used CAG, CAGGS, CASI, CBh, GFAP, TRE3G, SMVP, Spc512, H1/T0, CK7-miniCMV, pICAM2, HCRhAATp, and Tight promoters ( Table 2).

Chimeric gRNA expression is frequently driven by an RNA polymerase III promoter, most often by the human U6 small nuclear (U6) promoter. The human U6 promoter has been shown to be more efficient than its murine homolog for short hairpin RNA (shRNA) expression in both human and murine cells ^94^. The human U6 promoter was also proven more efficient than the human H1 promoter in driving shRNA expression for the long-term inhibition of gene expression *in vitro* and *in vivo* ^95^. However, the use of the U6 promoter to overexpress shRNAs *in vivo* can induce severe hepatotoxicity and inflammatory side effects due to abundant shRNA production ^96^. The weaker H1 promoter may offer a safer option in this case, but its therapeutic efficacy is dependent on the sequence of the shRNA ^96^. Some promoters require a particular nucleotide as the transcription start site (TSS) to initiate transcription. For instance, the human or mouse U6 promoters require a guanine nucleotide (G) at the 5′ end of the transcript for effective transcription ^97^. To use the U6 promoter for driving gRNA expression, the first nucleotide of the transcribed gRNA should be a G to maximize U6 promoter activity ^97^. If the G is absent at the TSS of the gRNA sequence, the human RNA polymerase III promoter H1 can be used to drive the expression of the gRNA ^76^. The use of H1 promoter-expressed gRNAs could enhance the versatility of the CRISPR technology by expanding the targetable genome ^98^. For multiplex CRISPR/Cas9-based genome editing such as large genomic DNA deletion or synergistically enhanced gene activation and repression, four independent polymerase III promoters (human U6 promoter, murine U6 promoter, 7SK, and H1) can be used to express four gRNAs in a single expression cassette ^21^. The use of distinct polymerase III promoters can avoid the problem of genetic recombination in a viral vector and the loss of the proximal gRNA due to identical DNA sequences of the promoters ^99^. The doxycycline-dependent promoters such as H1/TO and U6/TO can also be used for inducible *in vivo* genome editing by supplying animals with Dox-containing food to regulate the expression of the gRNA in a Dox-dependent manner ^81^. The introduction of Dox inhibits Tet repressor (TetR) binding and induces gRNA expression ^81^.

### Local and global delivery of AAV vectors

The route of administration of AAV-CRISPR vectors into the mouse body also determines the efficacy and specificity of gene editing for *in vivo* therapeutics ( Figure 1D). Given the inability of the most native AAV serotypes (except for AAV9 ^100^, AAVrh8 ^101^, and AAVrh10 ^102^) to cross the blood-brain barrier, stereotactic ^25, 65, 76^ and intrathecal ^84^ injections are still the preferred routes to systemic injection in order to introduce the AAV-CRISPR vectors into specific regions of the mouse brain or central nervous system for therapeutic gene editing. For the stereotactic injection, AAV-CRISPR vectors are intracranially injected into the brain by using a stereotaxic apparatus and the injection micropipette. In the intrathecal injection approach, AAV-CRISPR vectors are injected into the spinal canal or subarachnoid space to reach cerebrospinal fluid. For efficient therapeutic gene editing in the mouse muscle tissue, intramuscular (via tibialis anterior), intraperitoneal (via intraperitoneal space), or intravenous (via the tail vein, temporal vein, and retro-orbital) injections are the preferred methods to introduce AAV-CRISPR vectors, as have been demonstrated for the mdx mouse model of Duchenne muscular dystrophy ^55– 57^. Subretinal ^58, 60^ or intravitreal ^59, 85^ injections are used to deliver AAV-CRISPR vectors into the mouse retina for gene editing. As most of the AAV vectors will accumulate in the mouse liver tissue upon systemic injection, intravenous injection by tail vein or temporal vein can efficiently edit the genes associated with liver diseases ^4, 49, 71^. Systemic injection remains preferred to local injection in the liver tissue because the delivery is more uniform and the distribution of viral vectors is broader. It is also a good delivery mode for gene editing in multiple tissues, as it is almost non-invasive to the body. On the other hand, intracardiac ^64^, subcutaneous ^78^, intraperitoneal ^63^, or systemic ^62, 70^ injection has been used to introduce AAV9 vectors carrying CRISPR transgene into the mouse heart for gene editing. Intranasal and intratracheal injections are used to locally deliver the AAV-CRISPR vehicles into the mouse lung tissue ^65^. Therefore, the selection of an effective route of injections is dependent on the target tissue, AAV serotypes, and tissue-specific promoters used.

## CRISPR/Cas9-based human gene and cell therapies

In August 2016, the first human clinical trial using a CRISPR-based gene-editing technique was started for cancer immunotherapy of metastatic non-small cell lung cancer ^103^. Subsequently, 10 more early phase clinical trials are underway (ClinicalTrials.gov). One of these clinical trials has proceeded to phase 2 for the treatment of advanced esophageal cancer. Six of these early phase clinical trials are using the CRISPR/Cas9 system to create genetically altered immune cells, which are infused back into patients with an advanced stage of lung, bladder, prostate, renal, gastric, esophageal, or nasopharyngeal carcinoma or lymphoma to target and eradicate cancer cells ( Table 3). In general, the programmed cell death protein 1 gene ( *PDCD1*) will be knocked out in autologous T cells. These engineered T cells then will be selected and expanded *ex vivo* before infusion back into the patients. PDCD1, more commonly known as PD-1, is an inhibitory cell surface receptor involved in the regulation of T-cell function during immune response and tolerance. Knockout of *PD-1* in T cells extracted from the patient’s blood could prevent cells’ immune response from switch-off after re-introduction of the gene-edited cells into the patient’s bloodstream and attack and defeat the cancer cells ^104^. In fact, antibodies (for example, nivolumab and pembrolizumab) that neutralize PD-1 with high response and low adverse effect rates have been successfully used for cancer immunotherapy in human clinical trials ^105, 106^. Gene editing is expected to inhibit PD-1 with a greater certainty, and by multiplying the cells in the laboratory, scientists can enhance the efficacy of triggering an immune response against tumors ^104^.

**Table 3.** Ongoing human clinical trials involving CRISPR/Cas9-based gene and cellular therapies.

Condition	Intervention	Phase	Type	Primary objective and study design	Principle	Start date	Finish date	ClinicalTrials. gov identifier
Metastatic non- small cell lung cancer	Biological: CRISPR/Cas9- mediated *PD-1* knockout T cells from autologous origin Drug: cyclophosphamide, interleukin-2	Phase 1	*Ex vivo*	A dose-escalation study to evaluate the safety of *ex vivo* knocked-out, expanded, and selected *PD-1* knockout engineered T cells that are infused back into the patient for the treatment of metastatic non- small cell lung cancer	Target cancer cell	August 2016	April 2018	NCT02793856
Muscle-invasive bladder cancer stage IV	Biological: CRISPR/Cas9- mediated *PD-1* knockout T cells from autologous origin Drug: cyclophosphamide, interleukin-2	Phase 1	*Ex vivo*	A dose-escalation study of *ex vivo* knocked-out, expanded, and selected *PD-1* knockout engineered T cells that are infused back into the patient for the treatment of muscle-invasive bladder cancer	Target cancer cell	September 2016	September 2019	NCT02863913
Hormone-refractory prostate cancer	Biological: CRISPR/Cas9- mediated *PD-1* knockout T cells from autologous origin Drug: cyclophosphamide, interleukin-2	Phase 1	*Ex vivo*	A dose-escalation study of *ex vivo* knocked-out, expanded, and selected *PD-1* knockout engineered T cells that are infused back into the patient for the treatment of castration- resistant prostate cancer	Target cancer cell	November 2016	December 2020	NCT02867345
Metastatic renal cell carcinoma	Biological: CRISPR/Cas9- mediated *PD-1* knockout T cells from autologous origin Drug: cyclophosphamide, interleukin-2	Phase 1	*Ex vivo*	A dose-escalation study of *ex vivo* knocked-out, expanded, and selected *PD-1* knockout engineered T cells that are infused back into the patient for the treatment of metastatic advanced renal cancer	Target cancer cell	November 2016	November 2020	NCT02867332
Advanced esophageal cancer	Biological: CRISPR/Cas9- mediated *PD-1* knockout T cells from autologous origin Drug: cyclophosphamide, interleukin-2	Phase 2	*Ex vivo*	Evaluate the safety of *ex vivo* knocked-out, expanded, and selected *PD-1* knockout T cells that are infused back into the patient for the treatment of advanced esophageal cancer	Target cancer cell	March 2017	December 2018	NCT03081715
Gastric carcinoma stage IV, nasopharyngeal carcinoma stage IV, T-cell lymphoma stage IV, adult Hodgkin lymphoma stage IV, diffuse large B-cell lymphoma stage IV	Biological: CRISPR/Cas9- mediated *PD-1* knockout T cells from autologous origin Drug: fludarabine, cyclophosphamide, interleukin-2	Phase 1/2	*Ex vivo*	Evaluate the safety and clinical response of cell therapy using CRISPR-Cas9-mediated *PD-1* knockout EBV-CTL cells for the treatment of advanced-stage EBV-associated malignancies	Target EBV- associated cancer cell	April 2017	March 2022	NCT03044743
HIV-1-infection	Biological: CRISPR/Cas9- mediated *CCR5* modified CD34 ^+^ hematopoietic stem/ progenitor cells from donors Drug: anti-retroviral therapy	Phase 1	*Ex vivo*	Evaluate the safety and feasibility of allotransplantation with CRISPR/Cas9 *CCR5* gene modified CD34 ^+^ hematopoietic stem/progenitor cells in HIV-infected patients with hematological malignances	Target CCR5- positive immune cell	May 2017	May 2021	NCT03164135
B-cell leukemia, B-cell lymphoma	Biological: gene-disrupted allogeneic CD19-directed BBζ CAR-T cells (termed UCART019) will be generated by combining the lentiviral delivery of CAR and CRISPR RNA electroporation to disrupt endogenous *TCR* and *B2M* genes	Phase 1/2	*Ex vivo*	Evaluate the feasibility, safety, and *in vivo* persistence of UCART019 adoptively transferred T cells in patients with relapsed or refractory CD19 ^+^ leukemia and lymphoma	Target cancer cell	June 2017	May 2022	NCT03166878
Human papillomavirus- related malignant neoplasm	Biological: TALEN and CRISPR/Cas9	Phase 1	*In vivo*	An open-label and triple-cohort study to evaluate the safety and efficacy of TALEN and CRISPR/ Cas9 plasmids for the treatment of HPV persistency and HPV- related cervical intraepithelial neoplasia	Disrupt HPV E6/E7 DNA	January 2018	January 2019	NCT03057912
Neurofibromatosis type 1	Biological: establish isogenic *NF1* wild-type ( *NF1* ^+^/ ^+^), *NF1* heterozygous ( *NF1* ^+^/ ^−^), and *NF1* homozygous ( *NF1* ^−^/ ^−^) patient-specific iPSC lines using CRISPR/Cas9 technology	Phase 1	*Ex vivo*	Establish an iPSC bank for disease phenotypic characterization, drug screening, and identification that can reverse or alleviate the disease phenotypes	Collection of stem cells	November 2017	June 2019	NCT03332030
Gastrointestinal infection	Biological: knockout CRISPR and gain-of-function CRISPR SAM Procedure: duodenal biopsy	Phase 1	*Ex vivo*	Identify and establish a list of host cellular proteins that mediate norovirus infection in a stem cell- derived human intestinal enteroid model	Genome- wide genetic screening	January 2018	December 2020	NCT03342547
Sickle cell disease	Overall genetic literacy, CRISPR-specific literacy, and general attitudes and beliefs toward CRISPR	Observational	Cross- sectional	Study the attitudes, beliefs, and opinions of those with SCD, parents of those with SCD, and providers on the use of CRISPR/ Cas9 gene-editing	–	October 2017	June 2018	NCT03167450

Based on ClinicalTrials.gov database on human clinical trials performed in the US and worldwide. *B2M*, beta-2-microglobulin; CAR, chimeric antigen receptor; *CCR5*, C-C chemokine receptor type 5; CTL, cytotoxic T-lymphocyte; EBV, Epstein-Barr virus; HPV, human papillomavirus; iPSC, induced pluripotent stem cell; *NF1*, neurofibromatosis type 1; *PD-1*, programmed cell death protein 1 gene; SCD, sickle cell disease; TALEN, transcription activator-like effector nuclease; *TCR*, T-cell receptor.

These interventional studies aimed to evaluate the clinical response, safety, and maximal tolerant dose of the CRISPR-mediated *PD-1* knockout T cells in treating advanced-stage malignancies upon being infused back into patients. An anti-cancer chemotherapy drug, cyclophosphamide, will be administered for a few days to modify the immune micro-environment before cell infusion or to deplete regulatory T cells (Tregs) before collecting peripheral blood. After cell infusion, the interleukin-2 cytokine that has both immune-modulating and anti-tumor properties will be administered to sustain the survival of infused T cells. For cancer immunotherapy of lymphoma or leukemia, an anti-metabolite anti-cancer chemotherapy drug, fludarabine, will be co-administered with the cyclophosphamide before cell infusion. Biomarkers and immunological markers will be closely monitored after cell infusion to determine whether the injections are causing any serious adverse effects and to evaluate whether the patients will receive benefits from the treatment.

In another CRISPR/Cas9-based *ex vivo* gene therapy clinical trial, the CRISPR/Cas9 complex will be used to disrupt the C-C chemokine receptor type 5 ( *CCR5*) gene in the CD34 ^+^ hematopoietic stem or progenitor cells from donors (NCT03164135). Then, these *CCR5* modified CD34 ^+^ cells from donors will be infused into the HIV-infected patients with AIDS and hematological malignancies. In principle, HIV-1 virus requires CCR5 receptor on the surface of host immune cells (for example, T cells) to infect the cell; hence, disrupting or blocking the CCR5 receptor may make the immune cell resistant to the virus infection. When the *CCR5*-modified immune cells from the donor are mixed inside the body with the CCR5-positive immune cells from HIV-infected patients, the HIV-1 virus will infect and kill the CCR5-positive immune cells and the CCR5-negative immune cells will survive and eventually take over the immune function. The primary objectives of this clinical trial are to determine the safety of the allotransplantation of these CD34 ^+^ cells and to evaluate the resistance to the HIV-1 virus in HIV-infected patients after infusion of these CD34 ^+^ cells. Prior to the cell infusion, the patients will be treated with anti-retroviral therapy to achieve undetectable HIV-1 virus in the peripheral blood. During the follow-up, HIV-1 viral load, CD4 ^+^ T cells, and CCR5-negative cell counts in the peripheral blood will be monitored in HIV-infected patients.

Another promising *ex vivo* gene therapy clinical trial is the combined use of CRISPR/Cas9 gene-editing technology and chimeric antigen receptor (CAR)-based cancer immunotherapy (NCT03166878). The CAR-expressed T cells can be engineered to recognize leukemia antigens such as CD19 on B cells for the treatment of relapsed or refractory B-cell malignancies. However, the cost and technical difficulties in the production and expansion of CAR-expressed T cells have hindered the wide application of personalized autologous CAR–T cell therapy. Thus, universal CAR–T cells derived from healthy unrelated donors are developed to overcome some of these drawbacks. To evade host-mediated immunity and deliver anti-leukemic effects without graft-versus-host disease in patients with relapsed or refractory B-cell malignancies, gene-disrupted allogeneic CD19-directed BBζ CAR–T cells (known as UCART019) are developed by combining the lentiviral delivery of CAR and CRISPR mRNA electroporation to disrupt both of the endogenous T-cell receptor ( *TCR*) and beta-2 microglobulin ( *B2M*) genes. The TCR and B2M play important roles in triggering the immune response; hence, disrupting both of these genes could minimize immunogenicity associated with allotransplantation. The primary goal of these allogeneic CD19 CAR–T cells is to evaluate the feasibility, safety, and *in vivo* persistence of UCART019 adoptively transferred T cells in patients with relapsed or refractory CD19 ^+^ leukemia and lymphoma. The amount of UCART019 cells (engraftment), humoral host immunity, and anti-tumor response upon UCART019 cell infusions will be monitored.

Owing to the tissue complexities in humans, toxicity concern, and potential complications such as host immune response following a high dose of AAV vectors used to achieve significant therapeutic efficacy ^107^, the AAV-CRISPR viral delivery system is restricted to *ex vivo* gene editing or genetic manipulation in animals. One of the promising strategies to overcome this issue was to mutagenize the surface-exposed tyrosine residues on the AAV capsid in order to avoid AAV degradation by the host cell proteasome machinery and to improve AAV intracellular trafficking to the nucleus, which can lead to high transduction efficiency at lower vector doses ^107^. The combined use of capsid-modified and genome-modified next-generation AAV vectors has allowed higher transduction efficiency and transgene expression at further reduced doses ^108^. To date, the AAV-CRISPR gene-editing system was tested in non-human animals only, but the first *in vivo* gene therapy using CRISPR/Cas9 technology will be carried out in human clinical trials soon. In January 2018, an open-label and triple-cohort study will be conducted to evaluate the safety of therapeutic doses and the dosing regimen of CRISPR/Cas9 plasmid to treat human papillomavirus (HPV) persistency and HPV-related cervical intraepithelial neoplasia. Given the important roles that E6 and E7 play in HPV-driven carcinogenesis, CRISPR/Cas9-mediated disruption of HPV16 and HPV18 E6/E7 DNA could be an attractive approach for therapeutic interventions by significantly downregulating the expression of E6/E7 in order to induce HPV-associated cell apoptosis and to inhibit cell growth.

## Promises and hurdles associated with the AAV-CRISPR system for future clinical applications

An increasing number of studies in mice have clearly demonstrated that the combined use of tissue-specific minimal promoters, natural and engineered AAV serotypes, different routes of administration, and small Cas9 orthologues enables efficient packaging and precise delivery of AAV-CRISPR vectors for targeted *in vivo* genome editing in specific tissues with minimized side effects. Nevertheless, special considerations are required when selecting tissue-specific promoters, natural and engineered AAV serotypes, or routes of administration to avoid non-specific delivery and transgene expression of the AAV. In addition, the off-target effects, toxicity, and immunogenicity associated with CRISPR/Cas9 delivery remain to be fully resolved.

### Non-specific delivery and transgene expression

Tissue-specific promoters and AAV serotypes might still be able to transduce and induce transgene expression in healthy tissues or other non-target organs if a strong promoter or a high dose of viral vectors is introduced into the body by intravenous injection. Despite a significant improvement in the efficiency and specificity of newly discovered natural and engineered AAV variants for systemic delivery in mice, the toxicity and side effects associated with the non-specific delivery of the transgenes to the non-target tissues and organs remain a concern ^13, 89^. It is easier to get delivery vehicles taken up by liver than other organs upon systemic injection. Therefore, it is more challenging to specifically target non-liver organs than liver via a systemic delivery approach. For instance, recently discovered synthetic vectors, AAV-PHP.B ^13^ and tyrosine-mutant AAV ^89^, were found to transduce the adult mouse central nervous system more efficiently and widely than the natural AAV9 after intravenous injection. However, these synthetic vectors can also transduce the liver and other peripheral organs upon systemic delivery ^13, 89^. In this case, local injections such as stereotaxic injection into the brain can minimize the possible complications or adverse side effects associated with non-specific expression of CRISPR in healthy tissues or other non-target organs.

### Off-target effects

Even though CRISPR/Cas9 predominantly recognizes the intended target sites, a series of high-throughput genome-wide methods such as multiplex Digenome-seq ^109, 110^, ChIP-seq ^111, 112^, GUIDE-seq ^113^, and whole-genome sequencing ^114^ as well as targeted deep sequencing ^112^, Cas9 toxicity screens ^115^, and SITE-seq biochemical methods ^116^ have revealed evidence of off-target effects due to target mismatch tolerance of CRISPR/Cas9. As inter-individual natural genetic variation can affect CRISPR/Cas9 specificity ^117^, a recently developed CIRCLE-seq approach could be used to identify genome-wide off-target mutations of CRISPR/Cas9 that are associated with cell type-specific single-nucleotide polymorphisms to provide personalized specificity profiles ^118^. Notably, given the same target sequence of gRNA, off-target sites of Cas9 before and after being fused to a catalytic enzyme (for example, cytidine deaminase base editor and chromatin modifiers) could be different; therefore, independent assessment of their genome-wide specificities is recommended ^119^. Imprecise repair of Cas9-induced DNA double-stranded breaks can give rise to deleterious structural chromosomal rearrangements such as deletions, inversions, and translocations, which in turn may activate oncogenes or cause genome instability ^120^. Hence, high-throughput screenings of CRISPR/Cas9 off-target activity and further improvement in the fidelity of CRISPR/Cas9 on-target activity are essential for safety in clinical gene transfer applications. ****

There are many ways to minimize CRISPR/Cas9 off-target effects in the human genome. For example, a recently developed RNA-targeting Cas9 (RCas9) system could avoid permanent off-target genetic lesions in DNA-mediated CRISPR-based therapeutics ^121^. The RCas9 system consists of a fusion of rationally truncated dCas9 protein and PilT N-terminus (PIN) domain that can be packaged into the AAV vector for eliminating toxic microsatellite repeat expansion RNA or reducing repetitive RNA level without targeting the DNA ^121^. With a similar strategy, systemic delivery of dCas9/gRNA by AAV9 significantly reduced pathological RNA foci, rescued chloride channel 1 protein expression, and decreased myotonia in myotonic dystrophy mice by impeding the transcription of expanded microsatellite repeats ^122^. Another way is to deliver purified Cas9 RNPs rather than plasmid expression vectors ^10, 123^. Despite some technical constraints in non-viral delivery methods for *in vivo* administration, the use of Cas9 RNPs can limit the duration of Cas9 expression and decrease the chance of Cas9 nuclease cleaving at non-specific sites in a genome because of the rapid clearance from the cell ^10, 123^. Complementing the CRISPR-based editing capability with conditional genetic manipulation tools such as photoactivatable Cas9 ^124, 125^, chemical-inducible Cas9 ^126, 127^, or multiple inputs logic gate genetic circuits ^128, 129^ enables the precise spatial and temporal control of Cas9 activity inside the cell, which in turn leads to the reduction in off-target activity. Alternatively, a pair of Cas9 nickases ^130– 132^, dCas9-FokI ^133^, or high-fidelity Cas9 variants such as SpCas9-HF1 ^134^, eSpCas9 ^135^, and HypaCas9 ^136^ can be used to minimize undesired off-target mutagenesis.

The use of a scarless genome-editing strategy for targeted point mutation knock-in can also minimize unwanted mutation formations to favor the desired clean base editing outcomes ^137, 138^. For a point mutation knock-in, the efficiency of precise sequence replacement by CRISPR-mediated homology-directed repair (HDR) could be significantly increased by using asymmetric donor DNA ^139^, HDR enhancer ^140^, or short ssDNA donor oligonucleotides as a donor template instead of long plasmid donor ^139^ or by inhibiting non-homologous end joining (NHEJ) activity ^141^. Also, the artificial chimeric RNAs ^142^—truncated ^73, 113^ and chemically modified ^143^ gRNAs—were shown to have lower off-target activity than the original gRNAs. A number of bioinformatics analysis tools such as GuideScan ^144^, CRISPRdirect ^145^, and Cas-OFFinder ^146^ permit the specific design of gRNAs to avoid binding at the non-intended target sites in the human genome. The specificity of the gRNA designed can be improved by selecting a target sequence with two Gs at the 5′ terminus ^131^ and avoiding potential mismatches at the seed sequence or base-pairing adjacent to the PAM ^147^. Another innovative way to improve the specificity and reduce the toxicity of the CRISPR/Cas9 is co-delivery of DNA decoys or competitive inhibitor oligonucleotides bearing all possible off-target sequences that can sequester and prevent the CRISPR/Cas9 from binding to the off-target sites within a host genome. A similar concept has been successfully demonstrated to alleviate the off-target effects of RNA interference by using RNA decoys to reduce the sense strand activity of shRNAs ^148^, while artificial microRNA (miRNA) sponges have been used to inhibit miRNA function and its ability to regulate natural mRNAs ^149^. In addition, a recently developed miRNA-responsive CRISPR/Cas9 switch could be useful for cell type-specific genome editing by sensing endogenous miRNA activities ^150^.

### Immunogenicity

Owing to the exogenous nature of AAV and CRISPR components, host immune responses can attenuate therapeutic effects and cause side effects. Thus, the toxicity and immunogenicity associated with AAV and CRISPR components should be circumvented for safer and higher efficacy in clinical gene therapy applications. Even though the AAV generally elicits a very mild immune response and does not induce extensive cellular damage *in vivo*, certain AAV serotypes such as AAV9 may evoke humoral immune responses, as indicated by the presence of capsid-specific antibodies ^50^. While transient immunosuppression is one of the possible ways to mitigate the host immune response following the delivery of AAV vectors ^151^, it is not feasible for long-term therapeutic treatments of chronic diseases and is prone to adverse complications such as infections and malignancies. Empty capsid mutants can be used as decoys to overcome pre-existing humoral immunity by adsorbing antibodies in the bloodstream upon systemic delivery of both empty and functional AAVs ^152^. Alternatively, the AAV capsids can be genetically engineered or mutated to reduce the binding affinity and the neutralizing effects of AAV antibodies ^15, 153– 155^. In addition to AAVs, the presence of Cas9-specific antibodies in Cas9-exposed animals indicated that Cas9 could evoke deleterious cellular and humoral immune responses *in vivo* ^50^. Expression of Cas9 *in vivo* could affect the transcription of the genes associated with the immunological processes. This in turn may destabilize the host immune system, elicit significant cellular infiltration or expansion, and induce enlargement of the draining lymph nodes with increased immune cell counts ^50^. The Cas9-responsive T-cell clonotype described previously could serve as a distinctive biomarker to assess Cas9-specific immunity before clinical implementation of the CRISPR system ^50^. To minimize the immune response due to prolonged expression of Cas9, conditional genome editing with the self-limiting CRISPR/Cas9 system ^58^ or light-inducible ^124, 125^ or chemical-inducible ^126, 127^ Cas9 can be used to minimize the duration of Cas9 expression in the body.

## Conclusions and perspectives

The AAV-CRISPR system holds enormous translational potential to develop curative therapeutic options for patients with severe and life-threatening genetic diseases by permanently editing disease-causing or risk genes in the human body. The delivery, efficacy, and safety issues in treating complex heritable and somatic disorders have to be fully resolved to fulfill this promise. Thus far, the *ex vivo* approach has been adopted to overcome the technical challenges associated with the *in vivo* delivery of the AAV-CRISPR viral vectors in humans. The *ex vivo* gene-editing approach is commonly used for the therapeutic treatment of blood disorders, cancers, and immune-related diseases. In the near future, *in vivo* genome editing is expected to offer better avenues to treat a wide range of human hereditary diseases in adults. *In vivo* gene therapy in humans provides several advantages over the *ex vivo* approach, and sometimes a combination of the two is necessary to achieve a good therapeutic outcome. For example, *in vivo* photoreceptor cell rescue can be used to halt retinal degeneration to preserve existing vision, while *ex vivo* photoreceptor cell replacement can be used to restore lost vision in patients with retinal dystrophy ^156^. After *in vivo* photoreceptor cell rescue, the retinal environment may become more permissive for transplanted photo-receptor survival and *de novo* synaptogenesis via the *ex vivo* approach ^156^.

The AAV vector already has a long history of success in clinical trials *in vivo*, but owing to the relatively recent arrival of CRISPR technology, the AAV-CRISPR system has yet to be tested *in vivo* for human gene therapy trials. Nevertheless, three ongoing human clinical trials (NCT03041324, NCT02702115, and NCT02695160) have used AAV vectors to deliver zinc finger nucleases, an earlier and well-established gene-editing tool, to the liver tissue for the treatment of hemophilia B and mucopolysaccharidosis. Similarly, the AAV vectors may be used for CRISPR delivery for human clinical trials in the future. Excitingly, the first *in vivo* human gene therapy trials using CRISPR/Cas9 plasmid administration to treat human HPV-related malignant neoplasms will be initiated in January 2018 (NCT03057912).

Although it is more feasible to deliver the transgene *ex vivo* than *in vivo*, the *in vivo* approach eliminates the need for cell transplants. Therefore, *in vivo* therapy can avoid potential graft-versus-host disease and immunosuppression-related complications such as infections and malignancies, as seen in cellular therapy. Recently, various genetically engineered AAV variants or structure-guided derivations of AAV mutants have been developed to significantly improve the efficiency and specificity for *in vivo* delivery ^157^. Compared with existing AAV serotypes, these newly engineered AAV capsids enabled higher transduction efficiency at a targeted tissue by altering the native tropism of the AAV capsid ^13– 15, 158^ and had low immunogenicity by evading pre-existing anti-AAV capsid neutralizing antibodies in the human body ^159, 160^. Given the encouraging results obtained with the next-generation synthetic AAV capsids in animals, this synthetic AAV vector may also be used to deliver CRISPR/Cas9 transgene in humans for *in vivo* genome editing in the near future. Further improvement in the performance of engineered AAV variants and CRISPR components is necessary to realize the full potential of the AAV-CRISPR system for *in vivo* genome editing.
